# Supplementary material for: Serum Clusterin Concentration and Its Glycosylation Changes as Potential New Diagnostic Markers of SARS-CoV-2 Infection and Recovery Process
Source: Int J Mol Sci. 2024 Apr 10;25(8):4198. doi: 10.3390/ijms25084198 (PMC11049940; doi:10.3390/ijms25084198)
Supplement: Supplementary file 1 [file ijms-25-04198-s001.zip › ijms-2926399-supplementary.pdf]

Supplementary materials

Serum clusterin concentration and its glycosylation changes as potential new diagnostic markers of SARS-CoV-2 infection and recovery process

Katarzyna Sołkiewicz[1*], Izabela Kokot[1], Monika Kacperczyk[1], Violetta Dymicka-Piekarska[2], Justyna Dorf[2], Ewa Maria Kratz[1*]

[1] Department of Laboratory Diagnostics, Division of Laboratory Diagnostics, Faculty of Pharmacy, Wroclaw Medical University, Wroclaw, Poland

[2] Department of Clinical Laboratory Diagnostics, Medical University of Bialystok, Bialystok, Poland

Table S1. Modified Early Warning Score (MEWS)

Score / Parameters	3	2	1	0	1	2	3
**Respiratory rate** (breaths/min)		≤8		9–14	15–20	21–29	>29
**Heart rate,** (bpm)		≤40	41–50	51–100	101–110	111–129	>129
**Systolic blood pressure** (mm Hg)	≤70	71–80	81–100	101–199		≥200	
**Hourly urine,** (mL/kg of body weight/h)	Nil	<0.5					
**Body temperature** (°C)		≤35	35.1–36.0	36.1–38.0	38.1–38.5	≥38.6	
**Neurological symptoms**					Responsive to voice	Responsive to pain	Unresponsive

Table S2. The concentrations of blood serum HDL

Parameter / Groups	HDL concentration (mg/dL)
**COVID-19 n = 87**	46.37 ± 19.34
**CONVALESCENTS n = 50**	63.28 ± 17.56 ***p^1 = 0.000002***
**CONTROL n = 65**	66.27 ± 19.26 ***p^1 = 0.000000***

The concentrations of HDL (high-density lipoproteins) in sera were presented as mean values ± SD (standard deviation). Significant differences between the control group (healthy subjects) versus: [1]patients with severe COVID-19.
